# 基于金属有机骨架复合气凝胶的分散固相萃取-超高效液相色谱-串联质谱法测定水中5种非甾体类抗炎药

**DOI:** 10.3724/SP.J.1123.2021.07014

**Published:** 2022-04-08

**Authors:** Huijuan LING, Gege WU, Shuang LI, Qian ZHOU, Chunxin LI, Jiping MA

**Affiliations:** 1.青岛理工大学环境与市政工程学院, 山东 青岛 266033; 1. School of Environmental and Municipal Engineering, Qingdao University of Technology, Qingdao 266033, China; 2.光大青岛理工环境技术研究院, 山东 青岛 266033; 2. Everbright Qingdao Institute of Environmental Technology, Qingdao 266033, China

**Keywords:** 超高效液相色谱-串联质谱, 分散固相萃取, 金属有机骨架, 复合气凝胶, 非甾体抗炎药, 环境水体, ultra performance liquid chromatography-tandem mass spectrometry (UPLC-MS/MS), dispersive solid phase extraction (DSPE), metal-organic frameworks (MOFs), composite aerogel, nonsteroidal anti-inflammatory drugs (NSAIDs), environmental waters

## Abstract

非甾体类抗炎药(NSAIDs)能在环境水体中长期稳定存在,不仅对生物有慢性毒性还能增加病原体的耐药性,开发可靠的测定水样中痕量非甾体抗炎药的分析方法至关重要。该文制备新型金属有机骨架/壳聚糖复合气凝胶材料Co-UiO-67(bpy)/CS分散固相萃取吸附剂,将其用于环境水体中酮洛芬、萘普生、氟比洛芬、双氯芬酸、布洛芬5种非甾体类抗炎药的富集,结合超高效液相色谱-串联质谱法(UPLC-MS/MS),建立了基于金属有机骨架材料(MOFs)复合气凝胶环境水体中药物残留检测的新方法。为获得最佳的萃取效率,对影响萃取效果的主要因素(材料类型、MOFs用量、萃取时间、水样pH值、离子强度、甲酸体积分数、洗脱时间、洗脱剂体积)进行条件考察及优化。优化结果显示,吸附剂5 min内就可实现目标化合物的完全吸附,用总体积为5 mL的1%甲酸甲醇溶液洗脱6 min,目标化合物就能充分解吸。在最优的固相萃取条件下建立分析方法,结果表明,5种非甾体类抗炎药在各自范围内线性关系良好,线性相关系数均大于0.9937,方法的检出限(LOD)和定量限(LOQ)分别为0.32~2.06 ng/L和1.05~6.78 ng/L。在40、250和1500 ng/L 3个加标水平下进行加标回收试验,5种待测物的平均回收率为74.5%~114.1%。日内、日间相对标准偏差分别为1.3%~12.3%和1.3%~11.5%。将该方法用于实际水样的检测,市政污水检测出微量的酮洛芬和氟比洛芬,含量分别为14.52 ng/L和10.05 ng/L。该方法具有良好的灵敏度、准确度和精密度,操作简便,耗时短,为环境水体中痕量非甾体抗炎药富集检测提供了新方法。

## Abstract (en)

Nonsteroidal anti-inflammatory drugs (NSAIDs) are a class of synthetic drugs that do not contain glucocorticoids. NSAIDs are widely used for their analgesic, antipyretic, and anti-inflammatory effects. Due to their low adsorption coefficients and recalcitrance to biodegradation, NSAIDs readily enter environmental water through sewage discharge and exist stably for long periods. The long-term presence of trace amounts of NSAIDs in environmental water has adverse health effects on humans and animals. Therefore, it is important to establish an appropriately sensitive and reliable method for the determination of NSAIDs in environmental water, where their concentrations are low. Ultra performance liquid chromatography-tandem mass spectrometry (UPLC-MS/MS) is highly selective and sensitive, and so is especially suitable for detection of NSAIDs. Solid phase extraction is one of the most commonly used pretreatment methods. The extraction efficiency depends mainly on the adsorbents used. Metal-organic framework (MOF) aerogel SPE materials combine the attributes of highly selective adsorption property and high affinity. Moreover, the monolithic structure of the MOF aerogel composite simplifies the solid-liquid separation process. In this work, a novel MOF/chitosan (CS) composite designated Co-UiO-67(bpy)/CS, was prepared as the adsorbent material to enrich ketoprofen (KPF), naproxen (NPX), flurbiprofen (FPN), diclofenac (DCF), and ibuprofen (IBF) in water. This facilitated the detection of these compounds by UPLC-MS/MS. Co-UiO-67(bpy) was synthesized by a solvothermal method by mixing zirconium chloride, cobalt chloride, and the organic ligand 2,2-bipyridine-5,5 dicarboxylic acid. A CS suspension was used to prepared the hydrogel, which was freeze-dried to obtain the Co-UiO-67(bpy)/CS aerogel. The prepared material was characterized by Fourier transform-infrared spectroscopy (FT-IR) and scanning electron microscopy (SEM). Co-UiO-67 (bpy) was embedded into chitosan. A layered porous MOF composite aerogel was observed. The extraction efficiency of the five NSAIDs was investigated and optimized by assessing type of extraction material, MOF amount, extraction time, sample pH, ionic strength, formic acid concentration in eluent, elution time, and elution volume. The optimized results showed that the target compounds could be completely adsorbed within 5 min. In the UPLC-MS/MS experiment, NSAIDs were analyzed in the negative ionization multiple radiation monitoring (MRM) mode. Gradient elution was carried out with 0.01% formic acid aqueous solution and methanol as the mobile phases. The analytical method was established in the optimized extraction conditions. The five NSAIDs displayed good linearity with linear correlation coefficients greater than 0.9937. The limits of detection (LODs) and limits of quantification (LOQs) of this developed method were 0.32-2.06 ng/L and 1.05-6.78 ng/L, respectively. Satisfactory recoveries of the five analytes were achieved within 74.5%-114.1% at three spiked concentrations of 40, 250, and 1500 ng/L, as well as good precision with relative standard deviations of 1.3%-12.3% (intra-day) and 1.3%-11.5% (inter-day). The method was then used to test real-world water samples. Trace amounts of ketoprofen and flurbiprofen were detected in municipal wastewater (14.52 ng/L and 10.05 ng/L, respectively). The method exhibited good sensitivity, accuracy, and precision, and the operation process was convenient. The present study thus presents a novel method for the detection of the trace NSAIDs in environmental waters.

我国是药物生产、使用大国,非甾体类抗炎药(NSAIDs)是一类人工合成的不含糖皮质激素的药物。这类药物因具有镇痛、解热和抗炎等作用被广泛使用。非甾体抗炎药由于其吸附系数低、难以生物降解等特点,易通过污水排放进入环境水体,并在其中长期稳定存在,成为水中最常被检测到的药物^[1]^。大多数污水处理厂还没有建立药物残留的预防措施和监测手段,NSAIDs在污水处理厂没有得到有效去除就被释放到环境水体中^[2]^。研究发现,水体中痕量的NSAIDs对生物有慢性毒性,可增加病原体的耐药性,对人和其他动物的健康造成不利影响^[3]^。世界卫生组织(WHO)已将非甾体抗炎药视为新兴污染物^[4]^。因此,开发可靠的测定水样中非甾体抗炎药的分析方法至关重要。

目前非甾体类抗炎药检测方法主要有气相色谱法(GC)^[5]^、高效液相色谱法(HPLC)^[6]^、液相色谱-串联质谱法(LC-MS/MS)^[1]^、气相色谱-质谱法(GC-MS)^[7]^、毛细管电泳法(CE)^[8]^等。其中,超高效液相色谱-串联质谱法(UPLC-MS/MS)具有选择性高、检出限低的优点,特别适用于药物残留分析检测。NSAIDs在环境水体含量甚微,很难实现药物残留的直接准确测定,需要合适的样品前处理方法对其进行富集浓缩。常用的样品前处理方法有液相微萃取^[9]^、固相萃取^[10]^、固相微萃取^[11]^和磁固相萃取^[1]^等。

金属有机骨架(MOFs)是由有机配体和金属离子或团簇通过配位键连接而成的多孔晶体材料^[12]^。MOFs具有比表面积大及孔径尺寸可调等优点,常被选作固相萃取吸附剂。然而MOFs由于其粉末形态,作为吸附剂在样品前处理的应用中存在固液分离过程较为复杂等缺点。为了克服这一限制,可将MOFs与一些基底材料相结合^[13]^。一方面使得固液分离过程更加便捷,另一方面也可改善MOFs的理化性质。近年来,磁性MOFs^[14,15,16]^、MOFs膜^[16]^等MOFs复合材料成功应用于样品前处理领域,并发挥着越来越重要的作用。本课题组研制了不同种类的MOFs膜^[17,18,19]^及磁性MOFs材料^[20]^,并将其应用于水中农药的富集分析,开发了相关高效便捷的新型固相萃取技术。

气凝胶由Kistler^[21]^于1931年被首次提出,是通过冷冻干燥水凝胶这种具有三维交联网络结构的亲水高分子材料得到的,海绵体的气凝胶材料展现出了一些独特的结构特性,如孔隙度高、密度超低、质地柔软等,然而气凝胶的多孔结构并不具备良好的选择吸附性。将MOFs与气凝胶材料相结合,既保留了MOFs的高效选择吸附特性,具有多级孔径的MOFs气凝胶也增强了MOFs与目标物分子的亲和性,提高传质效率^[22]^。块状MOFs气凝胶也使得材料的固液分离过程更为简便。Yang等^[23]^以羧甲基纤维素(CMC)为基底,将制备的Ni/Co-MOF@CMC气凝胶用于盐酸四环素的去除,5 min内的去除效率约为80%。Fu等^[24]^通过冷冻干燥含有悬浮UiO-66纳米颗粒的壳聚糖水溶液,制备壳聚糖/UiO-66复合气凝胶作为水处理吸附剂,用于吸附水中甲基氯苯氧丙酸(MCPP)。该复合材料具有大孔结构,可增强传质速度,吸附完成后材料容易从溶液中回收。

本文采用双金属MOFs材料Co-UiO-67(bpy)、壳聚糖(CS)作为气凝胶基底通过冷冻干燥制得Co-UiO-67(bpy)/CS气凝胶。将其作为吸附剂,采用涡旋辅助分散固相萃取技术富集水中非甾体类抗炎药,结合超高效液相色谱-串联质谱法进行检测。

## 1 实验部分

### 1.1 仪器与试剂

QTRAP 3500超高效液相色谱-三重四极杆质谱仪(美国AB Sciex公司), Frontier傅里叶变换红外光谱仪(美国PerkinElmer公司), Sigma 300扫描电子显微镜(德国Zeiss公司),日本理学X射线粉末衍射仪(北京冠远科技有限公司), JTN200氮吹仪(杭州聚同电子有限公司), Vortex-2涡旋混匀仪(上海沪析实业有限公司), FreeZone冻干机(美国Labconco公司), SZCL-2数显智能控温磁力搅拌器(巩义市予华仪器有限责任公司), Millipore D-24UV超纯水机(美国Millipore公司)。

非甾体类抗炎药标准品:酮洛芬(KPF,纯度≥98%)、布洛芬(IBF,纯度≥98%)购自上海麦克林生化科技有限公司,萘普生(NPX,纯度≥99%)、双氯芬酸(DCF,纯度≥98%)、氟比洛芬(FPN,纯度≥98%)购自上海阿拉丁化学试剂有限公司。色谱纯甲醇购自德国默克公司,色谱纯甲酸购自天津科密欧试剂有限公司,环己烷购自国药集团化学试剂有限公司,*N*,*N*-二甲基甲酰胺(DMF)、冰醋酸、丙酮、戊二醛(GLA)购自天津富宇精细化工有限公司。

水样采集自某污水处理厂和某水库,所有水样经0.45 μm滤膜过滤后,于4 ℃保存于棕色玻璃瓶中。

### 1.2 材料的制备

按照文献^[25]^报道采用溶剂热法合成Co-UiO-67(bpy)。将氯化锆(0.048 g)、2,2-联吡啶-5,5二羧酸(0.045 g)、氯化钴(0.025 g)和冰醋酸(93 μL)溶于20 mL DMF中,超声混匀。将溶液转移到50 mL溶剂热反应釜中,并于120 ℃下反应48 h。通过离心收集得到Co-UiO-67(bpy),用DMF和甲醇清洗,并在烘箱中干燥12 h。

Co-UiO-67(bpy)/CS气凝胶的制备过程如图1所示。将壳聚糖(1 g)粉末溶解在2%醋酸水溶液中,得到质量分数为1%的壳聚糖溶液。然后取0.04 g Co-UiO-67(bpy)加入到50 mL烧杯中,加入10 mL质量分数为1%的壳聚糖溶液,再加入0.8 mL 0.5%戊二醛水溶液,于60 ℃水浴搅拌50 min,获得悬浊液。取1 mL悬浊液分装于小烧杯中,于-18 ℃冰箱中预冷冻12 h,再将其冷冻干燥24 h。取出后,用超纯水洗涤去除残留乙酸,用丙酮置换出气凝胶中的水,最后在环己烷中浸泡4 h,通风橱中风干,得到Co-UiO-67(bpy)/CS气凝胶。

**图1:**
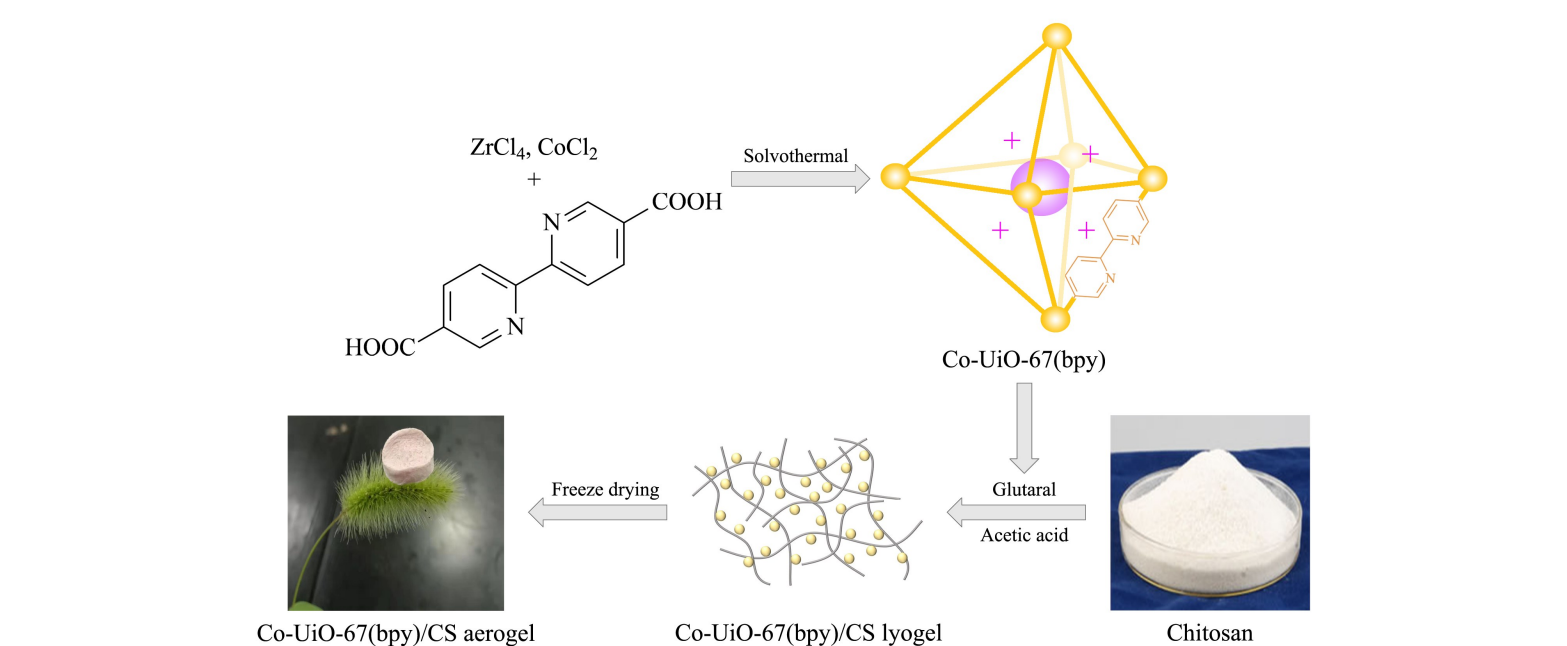
Co-UiO-67(bpy)/CS气凝胶的制备过程

### 1.3 样品前处理

将Co-UiO-67(bpy)/CS气凝胶材料置于50 mL离心管中,向其中加入20 mL水样,用涡旋混匀仪萃取5 min。萃取完成后,将Co-UiO-67(bpy)/CS气凝胶材料从水样中取出,用2.5 mL 1%甲酸甲醇溶液洗脱2次,每次3 min。收集的洗脱液氮吹浓缩至近干,用0.5 mL 35%甲醇水溶液复溶,用0.22 μm滤头过滤后使用UPLC-MS/MS检测。

### 1.4 仪器条件

采用ACQUITY UPLC BEH C18色谱柱(100 mm×2.1 mm, 1.7 μm;美国Waters公司);柱温40 ℃,流动相为(A)0.01%甲酸水溶液和(B)甲醇,流速为0.4 mL/min。梯度洗脱程序为:0~0.5 min, 65%A; 0.5~11.0 min, 65%A~5%A; 11.0~12.0 min, 5%A; 12.0~12.1 min, 5%A~65%A; 12.1~13.0 min, 65%A;。进样量为10 μL。

离子源:ESI源,负离子模式;多反应监测模式;离子化温度:350 ℃;电源电压:-4500 V,气帘气压力:2.07×10^5^ Pa;雾化气压力:3.45×10^5^ Pa;辅助器压力:4.14×10^5^ Pa。5种非甾体类抗炎药的其他质谱参数见表1。

**表1:** 5种非甾体类抗炎药的质谱参数

Analyte	t_R_/min	Precursor ion (m/z)	Product ions (m/z)	Declustering potentials/V	Collision energies/eV
Ketoprofen (KPF)	6.44	253.0	209.0^*^, 197.0	-36, -36	-8, -19
Naproxen (NPX)	6.73	229.3	169.8^*^, 184.8	-38, -38	-19, -19
Flurbiprofen (FPN)	8.10	243.1	198.9^*^, 178.7	-30, -30	-12, -12
Diclofenacacid (DCF)	8.42	294.3	249.8^*^, 213.7	-48, -48	-15, -15
Ibuprofen (IBF)	8.71	205.0	161.0^*^, 159.2	-40, -40	-12, -9

* Quantitative ion.

## 2 结果与讨论

### 2.1 材料的表征

图2为Co-UiO-67(bpy)和Co-UiO-67(bpy)/CS气凝胶材料的扫描电镜图。如图2a所示,制备的MOFs颗粒具有良好的分散性,MOFs颗粒的尺寸约为50 nm。

**图2:**
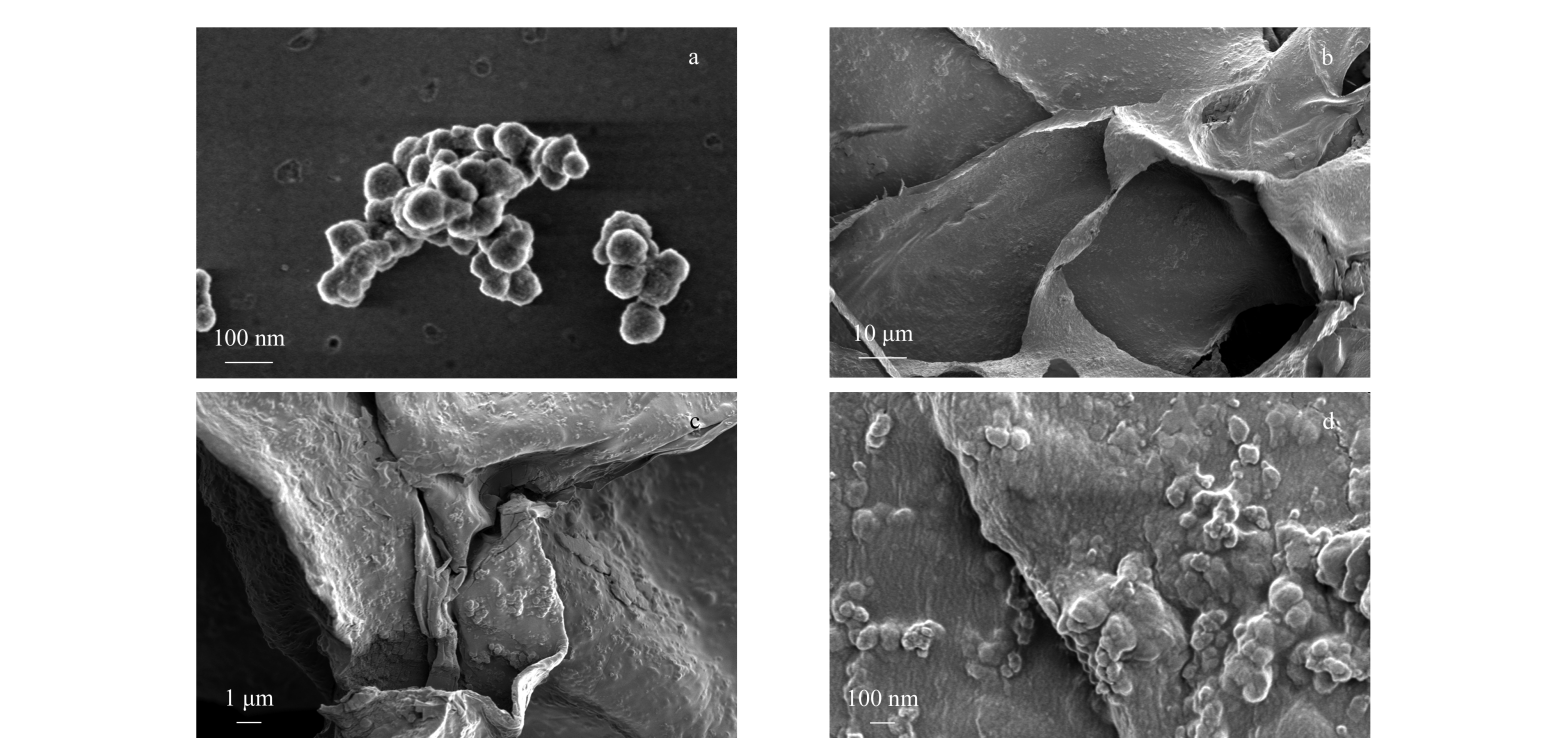
Co-UiO-67(bpy)和Co-UiO-67(bpy)/CS的扫描电镜图

如图2b和图2c所示,Co-UiO-67(bpy)/CS复合整体呈层状多孔结构,与文献^[26]^报道类似,存在冰晶升华连接的微米级孔。在富集在过程中,Co-UiO-67(bpy)/CS的分级大孔能够吸附大量的水,有利于固液传质^[27]^。在更高的放大倍数下(见图2d), Co-UiO-67(bpy)颗粒均匀分布在气凝胶的表面,部分颗粒嵌入壳聚糖孔壁^[24]^。

如图3a Co-UiO-67(bpy)的红外光谱图所示,在1609 cm^-1^处出现了吡啶环的伸缩振动峰,1460 cm^-1^处出现了C=N的伸缩振动峰。位于1250 cm^-1^和1141 cm^-1^的吸收峰为C-N伸缩振动峰和N-H的弯曲振动峰^[28,29]^,这些结果表明联吡啶配体存在于Co-UiO-67(bpy)中。如图3b所示,Co-UiO-67(bpy)/CS复合整体不仅具有MOFs类似的特征峰,在3451、1632、1082和1023 cm^-1^处也有明显的吸收峰,其中3451 cm^-1^处的吸收峰由壳聚糖侧链氨基N-H伸缩振动和羟基的O-H伸缩振动产生^[30]^。新出现的1632 cm^-1^处的峰是C=N的伸缩振动峰,证实了CS上的氨基可以与戊二醛反应形成亚氨基^[31]^。表明合成的Co-UiO-67(bpy)/CS材料为壳聚糖与Co-UiO-67(bpy)掺杂而成的复合整体。

**图3:**
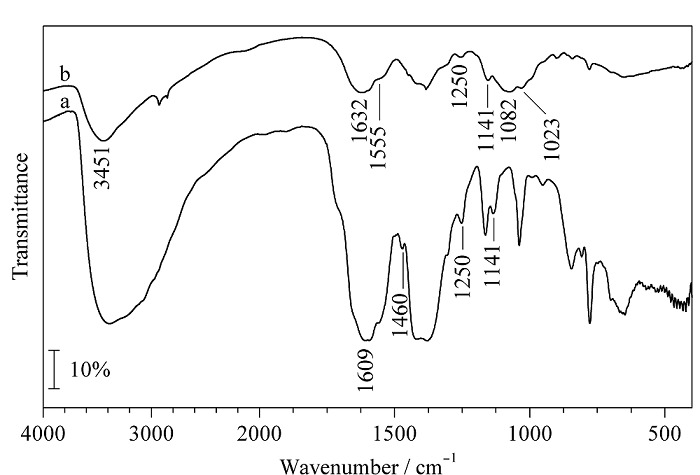
(a) Co-UiO-67(bpy)和(b) Co-UiO-67(bpy)/CS的红外光谱图

使用X射线衍射(XRD)对制备的Co-UiO-67(bpy)及Co-UiO-67(bpy)/CS复合材料的晶体结构进行表征。如图4a所示,Co-UiO-67(bpy)的XRD图谱与UiO-67的图谱非常吻合,在2*θ*为5.3°、9.3°、11.2°及17.2°出现的特征峰在与文献^[32]^报道一致。MOFs粉末衍射峰的信号较弱,可能是合成的MOFs粉末粒径较小。图4b为复合气凝胶的XRD图,与MOFs粉末具有一致的特征峰。

**图4:**
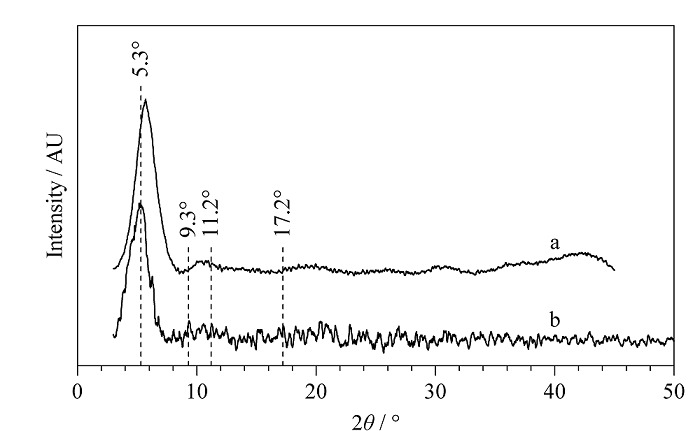
(a) Co-UiO-67(bpy)和(b) Co-UiO-67(bpy)/CS的X-射线衍射图

### 2.2 色谱条件的优化

比较了甲醇-0.01%甲酸水溶液和乙腈-0.01%甲酸水溶液作为流动相时目标化合物的分离效果。结果表明,使用甲醇作为有机相时,5种目标化合物的分离效果较好,因此选择甲醇-0.01%甲酸水溶液作为流动相。5种非甾体类抗炎药的总离子流色谱图见图5。

**图5:**
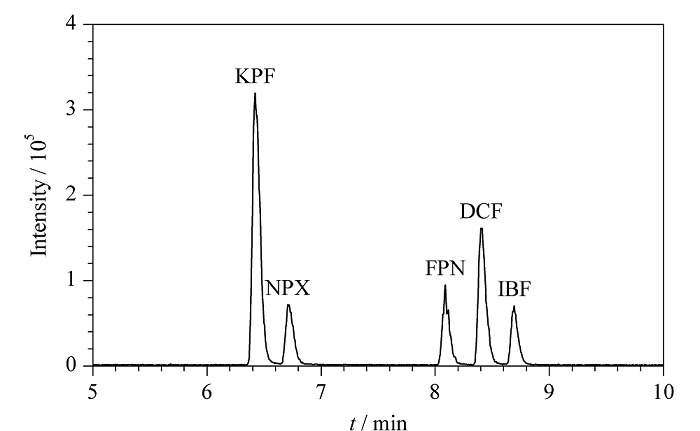
5种非甾体类抗炎药的总离子流色谱图

### 2.3 前处理条件的优化

为获得最佳的萃取效率,对材料类型、MOFs用量、萃取条件(如萃取时间、水样pH值和离子强度)、洗脱条件(如甲酸体积分数、洗脱时间、洗脱剂体积)进行优化考察。

2.3.1 材料类型

本实验对比Co-UiO-67(bpy)粉末、纯壳聚糖气凝胶和Co-UiO-67(bpy)/CS气凝胶3种材料对水中5种NSAIDs的萃取性能。如图6a所示,Co-UiO-67(bpy)粉末作为萃取吸附剂,其萃取效果并不理想,可能是因为吸附了目标化合物的MOFs粉末分散在溶液中,通过离心回收会损失部分MOFs。因此考虑用壳聚糖包裹MOFs使固液分离,过程更加便捷。纯壳聚糖气凝胶也能富集水中NSAIDs,将粉末MOFs掺杂进壳聚糖制作复合气凝胶,能进一步提高萃取效率,酮洛芬的萃取效率提高的最为明显,原因可能是Co-UiO-67(bpy)/CS这种复合材料能将MOFs粉末与气凝胶的优点相结合,使其表现出最佳的萃取效果。因此,采用Co-UiO-67(bpy)/CS萃取富集水中NSAIDs。

**图6:**
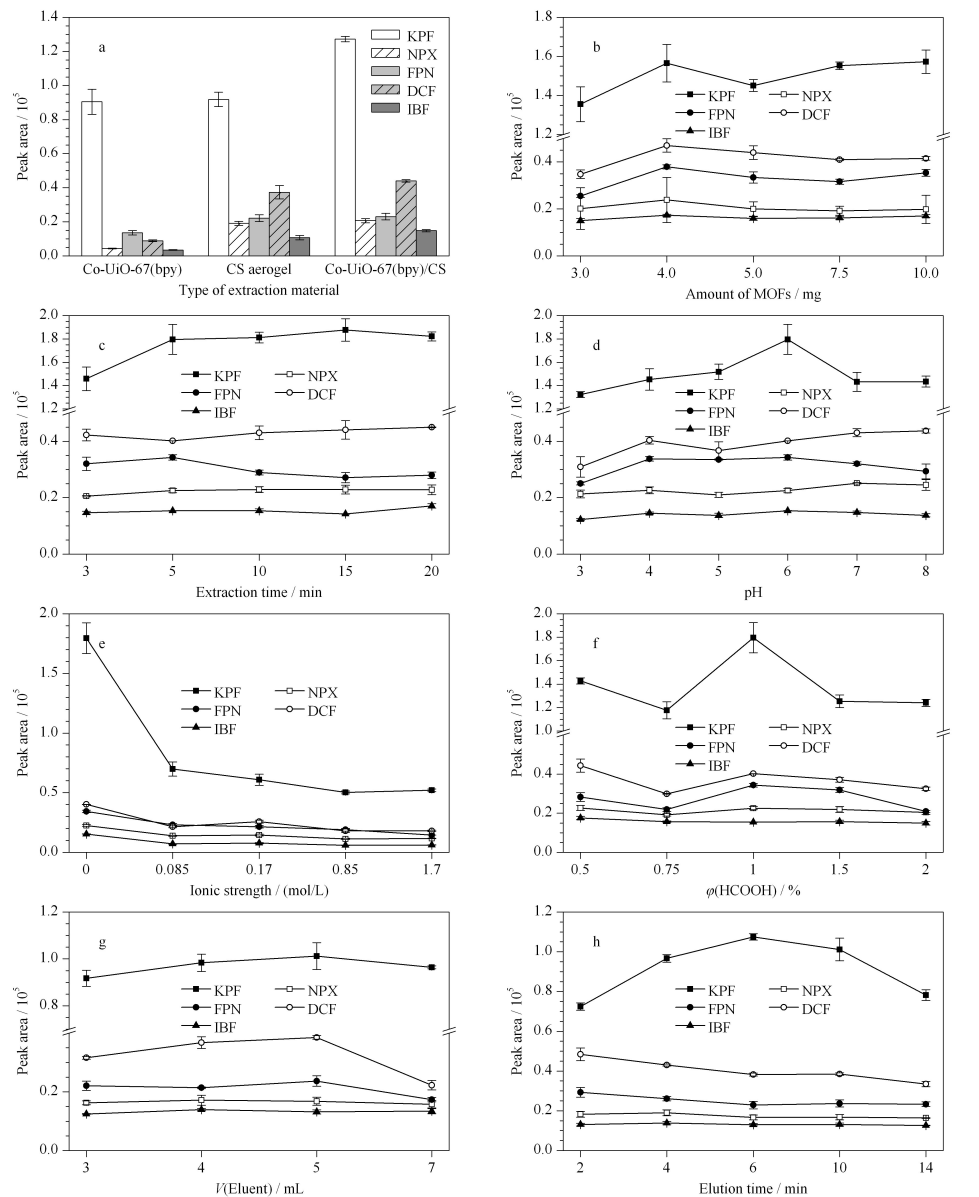
(a)材料类型、(b)MOFs用量、(c)萃取时间、(d)样品pH值、(e)离子强度、(f)甲酸体积分数、 (g)洗脱剂体积和(h)洗脱时间对5种非甾体类抗炎药萃取效果的影响(*n*=3)

2.3.2 MOFs用量

实验分别考察MOFs用量为3.0、4.0、5.0、7.5和10.0 mg时的萃取效果。如图6b所示,MOFs用量增加至4.0 mg时,5种NSAIDs的萃取效率最高,随后增加MOFs用量,萃取效率略有下降。这一现象可能是由于过量的MOFs导致Co-UiO-67(bpy)在壳聚糖基底上分布不均匀,阻碍了其对水中目标分析物的萃取。因此,在随后的研究中MOFs用量设为4.0 mg。

2.3.3 萃取时间

充分的萃取时间可使吸附剂捕获目标分析物直至萃取平衡。选择3~20 min的范围来考察萃取时间对萃取效率的影响。如图6c所示,当萃取时间为5 min时,FPN的萃取效率达到最高,延长萃取时间,萃取效率呈下降趋势。其他4种目标分析物萃取时间由3 min增加到5 min时,萃取效率明显提高,继续延长萃取时间萃取效率无明显变化。因此5 min作为最佳萃取时间。

2.3.4 水样pH值

水样的pH值不仅影响目标分析物在水中的存在形式,还影响Co-UiO-67(bpy)/CS的表面电荷。实验考察水样pH值为3、4、5、6、7和8时NSAIDs的萃取效率,结果如图6d所示。在pH 6时,5种NSAIDs的萃取效率总体达到了最高。这可能是因为5种NSAIDs的p*K*_a_为4.15~4.45,当溶液的pH小于4.5, NSAIDs在水溶液中带有正电荷或为中性分子形式存在,而双金属MOFs材料带有正电性,壳聚糖基底中的-NH_2_同样呈现正电性,材料与NSAIDs静电作用较弱导致萃取效率较低。而在碱性条件下,溶液中存在的阴离子OH^-^会对带有正电性双金属MOFs材料产生竞争吸附,导致萃取效率较低。超纯水pH值约为6,因此选择不调节水样pH值进行后续实验。

2.3.5 离子强度

溶液中共存的离子强度可能会影响吸附剂对目标分析物的亲和力。调节水样中氯化钠浓度(0、0.085、0.17、0.85、1.7 mol/L)来研究离子强度对萃取效果的影响。结果如图6e所示,可以看出随着离子强度的增加,萃取效率明显呈下降趋势,可能是因为解离的NaCl分子在溶液中产生了自由Cl^-^,与带有正电性双金属MOFs材料产生表面竞争吸附,影响其萃取效果。因此,在后续实验中选择不添加NaCl。

2.3.6 甲酸的体积分数

甲醇作为一种极性较强的有机溶剂,可以将NSAIDs洗脱下来,同时,在有机溶剂中加入适当甲酸可以提高极性分析物的洗脱效率。本实验采用体积分数为0.5%、0.75%、1%、1.5%、2%的甲酸甲醇溶液作为洗脱剂,考察其对萃取效率的影响。结果如图6f所示,甲酸体积分数为1%时,5种NSAIDs的萃取效率最高,但继续增加甲酸的体积分数,萃取效率明显呈下降趋势,可能是过量的甲酸破坏了Co-UiO-67(bpy)/CS的结构,使萃取效率下降。因此,选用1%甲酸甲醇溶液作为洗脱剂。

2.3.7 洗脱剂体积

洗脱剂体积影响目标分析物从吸附材料上解吸下来的程度。考察了单次洗脱剂体积分别为1.5、2.0、2.5、3.5 mL,同时洗脱步骤各进行2次时的洗脱效果。如图6g所示,总洗脱剂体积为5 mL时萃取效率最高,因此选为所用。

2.3.8 洗脱时间

在2~14 min范围内优化洗脱时间。如图6h所示,洗脱时间为6 min时,5种NSAIDs的萃取效率较高,此时目标化合物能充分解吸,因此后续实验选择6 min作为洗脱时间。

### 2.4 方法学验证

2.4.1 线性范围、检出限和定量限

将标准储备液连续稀释获得7个不同浓度水平(5、10、100、200、500、1000、2000 ng/L)的溶液,采用以上建立的分析方法对5种NSAIDs进行富集检测。以峰面积为纵坐标、质量浓度为横坐标绘制5种NSAIDs的标准曲线。结果如表2所示,检出限(LOD)和定量限(LOQ)分别通过3倍和10倍信噪比获得,5种NSAIDs的相关系数(*r*^2^)为0.9937~0.9993,检出限为0.32~2.06 ng/L,定量限为1.05~6.78 ng/L。

**表2:** 5种非甾体类抗炎药的线性方程、相关系数、线性范围、检出限和定量限

Analyte	Linear equation	r^2^	Linear range/(ng/L)	LOD/(ng/L)	LOQ/(ng/L)
KPF	y=3.89×10^2^x+1.68×10^3^	0.9986	10-2000	0.32	1.05
NPX	y=7.07×10^2^x+3.52×10^2^	0.9982	5-2000	2.06	6.78
FPN	y=9.34×10^1^x+6.17×10^2^	0.9964	10-2000	1.21	3.97
DCF	y=1.27×10^2^x+2.01×10^3^	0.9937	5-2000	0.70	2.32
IBF	y=4.71×10^1^x+7.74×10^2^	0.9993	10-2000	1.51	4.97

*y*: peak area; *x*: mass concentration, ng/L.

2.4.2 回收率和精密度

取空白水样,添加NSAIDs标准品,使得低、中、高加标水平分别为40、250和1500 ng/L。通过每个浓度点1 d内测定6个平行样考察日内精密度,每个浓度点连续测定6 d考察日间精密度。结果如表3所示,5种非甾体类抗炎药的加标回收率为74.5%~114.1%,日内日间精密度分别为1.3%~12.3%和1.3%~11.5%。

**表3:** 5种非甾体类抗炎药的加标回收率和精密度(*n*=6)

Analyte	Spiked/ (ng/L)	Recovery/ %	RSDs/%	
			Intra-day	Inter-day
KPF	40	78.9	8.8	11.0
	250	107.9	9.4	8.6
	1500	89.1	6.6	11.2
NPX	40	82.9	7.1	1.3
	250	87.0	3.8	10.6
	1500	92.6	3.3	9.8
FPN	40	114.1	5.1	6.1
	250	100.4	10.7	9.2
	1500	101.0	5.3	6.6
DCF	40	74.5	2.5	11.5
	250	97.8	11.7	8.3
	1500	104.7	12.3	10.3
IBF	40	79.7	1.3	4.5
	250	83.7	5.0	8.7
	1500	89.6	6.1	8.8

### 2.5 与文献方法对比

将建立的分析方法与报道的检测器灵敏度相似的水体中非甾体类抗炎药的分析方法进行比较(见表4)。本方法将分散固相萃取吸附剂制备成气凝胶形式,材料不需要通过离心、过滤,只需简单操作就可实现固液分离。与现有的分析方法相比,当前方法的萃取时间较短,仅为11 min。此外,该方法不需要调整水样pH值,简化了前处理步骤。该方法不仅可以用于文献报道中常用的地表水样的分析,还可以应用于复杂水样如市政污水的分析,表明Co-UiO-67(bpy)/CS对NSAIDs具有很好的选择性。所建立分析方法的检出限低于文献报道的NSAIDs的检出限。

**表4:** 本方法与文献报道的非甾体类抗炎药分析方法比较

Material	Method	Matrices	Extraction time/min	Practicable pH	LOD/ (ng/L)	Ref.
Fe_3_O_4_@/MIL-101(Cr)	MSPE-UPLC-MS/MS	waste water	18	5	3.00-60.00	[1]
MIL-101(Cr)/PVA	VA-SPE-HPLC-MS/MS	river, pharmaceutical,	60	4	7.00-37.00	[33]
		waste and feed water				
Fe_3_O_4_@MIL-100(Fe)	MSPE-UPLC-MS/MS	feed, waste, lake and	60	5	20.00-90.00	[34]
		pharmaceutical water				
C18	SPE-HPLC-MS/MS	surface water	>80	3	0.01-0.07	[35]
MIL-101(Cr)@GA	SPE-UPLC-MS/MS	river, lake and waste water	30	4	6.00-12.00	[36]
PANI/Pan NFsM	SPE-UPLC-MS/MS	drinking water	>20	7	0.40-5.00	[37]
Co-UiO-67(bpy)/CS	DSPE-UPLC-MS/MS	reservoir and waste water	11	6	0.32-2.06	this work

MIL-101(Cr)/PVA: MIL-101(Cr) polyvinyl alcohol cryogel; GA: graphene aerogel; PANI/Pan NFsM: core-shell polyaniline/polyacrylonitrile nanofibers mat; MSPE: magnetic solid phase extraction; VA-SPE: vortex assisted solid phase extraction; DSPE: dispersive solid phase extraction.

### 2.6 实际样品分析

对水库水和市政污水样品进行分析,结果如表5所示,水库水中没有检测到NSAIDs的存在,市政污水中检测到微量的酮洛芬和氟比洛芬,含量分别为14.52 ng/L和10.05 ng/L。水库水样中5种NSAIDs的回收率为74.6%~113.1%,市政污水样品回收率为69.7%~104.7%。上述结果表明该方法对水样中5种非甾体类抗炎药的富集和测定具有较高的实用性。

**表5:** 实际水样中5种非甾体类抗炎药的分析结果(n=3)

Analyte	Spiked/ (ng/L)	Reservoir water				Waste water		
		Found/(ng/L)	Recovery/%	RSD/%		Found/(ng/L)	Recovery/%	RSD/%
KPF	0	ND				14.52		
	40	44.25	110.6	11.9		55.85	103.3	12.6
	250	243.70	93.9	10.2		259.51	98.0	7.9
	1500	1103.83	73.6	6.9		1060.06	69.7	0.1
NPX	0	ND				ND		
	40	42.47	106.2	6.8		37.23	93.1	2.1
	250	203.12	81.3	5.9		201.08	80.4	7.3
	1500	1524.74	101.7	7.7		1253.34	83.6	4.8
FPN	0	ND				10.05		
	40	45.24	113.1	6.0		42.47	81.0	3.1
	250	239.73	95.9	2.5		250.77	96.3	3.9
	1500	1525.16	101.7	5.9		1403.86	92.9	12.0
DCF	0	ND				ND		
	40	33.60	84.0	2.8		33.97	84.9	5.6
	250	190.50	76.2	5.4		219.08	87.6	5.4
	1500	1488.03	99.2	10.9		1291.94	86.1	1.3
IBF	0	ND				ND		
	40	37.32	93.3	1.3		41.89	104.7	5.3
	250	186.57	74.6	1.4		193.62	77.4	9.3
	1500	1341.46	89.4	0.6		1108.73	73.9	2.4

ND: not detected.

## 3 结论

本文制备出一种新型的Co-UiO-67(bpy)/CS气凝胶材料,该材料不仅具有MOFs的高效选择吸附特性,也使固液分离更为简便。将其作为分散固相萃取吸附剂,能实现水中超痕量非甾体类抗炎药的高效富集,结合UPLC-MS/MS进行检测,建立了分散固相萃取-超高效液相色谱-串联质谱测定水中5种非甾体类抗炎药的方法。该方法的灵敏度、准确度满足实际样品检测要求,为水中其他有机污染物的富集检测提供了新的思路。
